# Sex differences in soluble prorenin receptor in patients with type 2 diabetes

**DOI:** 10.1186/s13293-021-00374-3

**Published:** 2021-05-01

**Authors:** Bruna Visniauskas, Danielle Y. Arita, Carla B. Rosales, Mohammed A. Feroz, Christina Luffman, Michael J. Accavitti, Gabrielle Dawkins, Jennifer Hong, Andrew C. Curnow, Tina K. Thethi, John J. Lefante, Edgar A. Jaimes, Franck Mauvais-Jarvis, Vivian A. Fonseca, Minolfa C. Prieto

**Affiliations:** 1 Department of Physiology, Tulane University School of Medicine, 1430 Tulane Avenue, SL39, New Orleans, LA 70112 USA; 2 Department of Medicine, Endocrinology Division, Tulane University School of Medicine, New Orleans, LA USA; 3 AdventHealth, Translational Research Institute, Orlando, FL USA; 4 Department of Biostatistics and Data Science, School of Public Health and Tropical Medicine, New Orleans, LA USA; 5 Renal Service, Memorial Sloan Kettering Cancer Center, New York, NY USA; 6 Southeast Louisiana Veterans Healthcare System, New Orleans, LA USA; 7 Tulane Center of Excellence in Sex-Based Biology and Medicine, New Orleans, LA USA; 8 Tulane Hypertension and Renal Center of Excellence, Tulane University School of Medicine, New Orleans, LA USA

**Keywords:** Plasma renin activity, Urine renin activity, eGFR, Urine angiotensinogen, Sexual dimorphism

## Abstract

**Background:**

The soluble prorenin receptor (sPRR), a member of the renin-angiotensin system (RAS), is elevated in plasma of patients with preeclampsia, hypertension, chronic kidney disease (CKD), and type 2 diabetes. Our goal was to examine the relationship between sPRR and RAS activation to define whether sexual dimorphisms in sPRR might explain sex disparities in renal outcomes in patients with type 2 diabetes.

**Methods:**

Two hundred sixty-nine participants were included in the study (mean age, 48 ± 16 years; 42% men, 58% women), including 173 controls and 96 subjects with type 2 diabetes. In plasma and urine, we measured sPRR, plasma renin activity (PRA), and prorenin. In the urine, we also measured angiotensinogen along with other biomarkers of renal dysfunction.

**Results:**

Plasma sPRR and PRA were significantly higher in women with type 2 diabetes compared to men. In these women, plasma sPRR was positively correlated with PRA, age, and body mass index (BMI). In contrast, in men the sPRR in urine but not in plasma positively correlated with eGFR in urine, but negatively correlated with urine renin activity, plasma glucose, age, and BMI.

**Conclusions:**

In patients with type 2 diabetes, sPRR contributes to RAS stimulation in a sex-dependent fashion. In diabetic women, increased plasma sPRR parallels the activation of systemic RAS; while in diabetic men, decreased sPRR in urine matches intrarenal RAS stimulation. sPRR might be a potential indicator of intrarenal RAS activation and renal dysfunction in men and women with type 2 diabetes.

**Supplementary Information:**

The online version contains supplementary material available at 10.1186/s13293-021-00374-3.

## Introduction

Type 2 diabetes mellitus is a major risk factor for cardiovascular (CV) and renal diseases [1]. A high percentage of patients with type 2 diabetes exhibit inappropriate activation of systemic and intrarenal renin-angiotensin systems (RAS) [2], which increases the risk of acute coronary events and accelerate the progression of diabetic nephropathy (DN) to chronic kidney disease (CKD) [3], particularly in menopausal women with type 2 diabetes [4]. Identifying a novel candidate biomarker that reflects systemic and intrarenal RAS status is crucial for reducing the prevalence of CV events and the progression of DN.

In 2002, Nguyen et al. [5] cloned the prorenin receptor (PRR), a single transmembrane domain protein that fully activates prorenin and enhances the catalytic activity of renin [6, 7]. PRR-dependent activation of prorenin catalyzes the conversion of angiotensinogen (AGT) to angiotensin-I (Ang-I), which is a key process in the RAS cascade [5]. Furthermore, the binding of prorenin to PRR stimulates tissue profibrotic genes, independent of the RAS [8, 9]. In humans, rats, and mice, PRR is expressed in various tissues, including the brain, heart, fat, blood vessels, placenta, lung, and kidney [5, 10–12]. In the kidney, PRR is upregulated in diabetes [13] and its inhibition ameliorates DN [14, 15]. Besides a membrane-bound isoform, the PRR exhibits a soluble isoform (sPRR) that is processed intracellularly by various serine proteases [16–18]. The levels of sPRR are increased in the plasma of patients with essential hypertension, preeclampsia, gestational diabetes, obstructive sleep apnea, DN, and CKD [19–22]. However, the relationship between sPRR and renal dysfunction in patients with type 2 diabetes remains unclear.

In patients with type 2 diabetes, high circulating levels of prorenin are associated with the onset of microalbuminuria [23] and the onset of microvascular complications [24, 25]. The ability of sPRR to activate prorenin in the extracellular space raises the possibility that the interactions between sPRR and prorenin in the systemic circulation, or within the renal intratubular compartments, may contribute to either systemic or intrarenal RAS activation and the progression of CKD in patients with type 2 diabetes [11, 26]. Sex also influences fasting glucose and glucose tolerance responses through gonadal hormones controlling glucose homeostasis [27]. In type 2 diabetes, dysregulation of the RAS contributes to the development of diabetic kidney disease and CV complications. While premenopausal women are protected from these complications, the protection weakens after menopause and even more in those with type 2 diabetes [28, 29]. However, it is unknown whether sex differences in sPRR concentrations in plasma and/or urine are associated with renal dysfunction in patients with type 2 diabetes. In the present study, we examined the effects of sex on plasma and urine sPRR concentrations, and their relationship with systemic RAS activation and decline in renal function in a cohort of men and women with type 2 diabetes.

## Material and methods

### Study participants

This was a randomized and cross-sectional study that enrolled 269 individuals at Tulane University Health Sciences Center and all participants were enrolled from November 2008 to June 2012. The inclusion criteria were male or female of age > 18 years old. Participants that self-reported history of drug abuse or pregnancy were excluded from the study. During the first visit, a complete medical history was obtained, and a physical exam was performed. From each patient, 5 mL of fasting blood was drawn into EDTA-containing tubes, placed on ice, centrifuged within 10 min in a refrigerated centrifuge at 1300×*g* for 10 min, to collect plasmas which were then snap frozen in liquid nitrogen, and stored in − 80 °C freezer until assayed. The time interval between blood sampling and centrifugation was minimized to prevent false positive increases in sPRR levels as well as renin activity. In patients with history of type 2 diabetes, hemoglobin A1c (HbA1c) was measured as well. Similarly, quickly after urine collection, samples were centrifuged at 1300×*g* at 4 °C for 10 min, and then stored in − 80 °C freezer until assayed

### Background factors and laboratory data at enrollment

Age, sex, race, BMI, waist-to-hip ratio (WHR), fasting blood glucose, blood pressure, and plasma creatinine levels were measured using standard laboratory methods at the Tulane Clinical Science Center. The estimated glomerular filtration rate (eGFR) was calculated using a prediction equation developed from the Modification of Diet in Renal Disease study [30].

The concentration of sPRR measured by ELISA (#27782, IBL America, Minneapolis, MN) and renin activity by radioimmunoassay (#CA1553, DiaSorin Inc, Stillwater, MN). In the urine, we quantified the concentrations of creatinine and albumin to creatinine ratio (ACR) (DCA-2000, Bayer AG, Germany), and angiotensinogen (uAGT) and transforming growth factor-beta 1 (TGF-β1) concentrations were quantified by ELISA kits (#24412, IBL America; #MB100B, R&D Systems, Minneapolis, MN). Urine samples were concentrated ten-fold using Amicon Ultra-10 centrifugal filters (Millipore, Massachusetts, USA) before the quantification of prorenin (#IHPREN-HIS, Molecular Innovations, Novi, MI).

### Statistical analyses

The values were expressed as means and SD or 95% confident intervals for continuous variables. The cohort was divided into 2 groups: control group (participants with no personal history of diabetes) and patients with type 2 diabetes (for those with history of type 2 diabetes). One-way analysis of variance followed by Bonferroni comparisons and unpaired *t*-tests were used to compare continuous variables between groups. Pearson correlation coefficients were performed to determine associations between plasma, urine sPRR, and the background factors. The relationship between eGFR (a continuous outcome) and sPRR variables was evaluated using multiple linear regression models. The levels of significance were defined as *P* < 0.05. All analyses were performed using SAS v9.4.

## Results

### Demographic characteristics of the study participants

Table 1 shows demographic and clinical characteristics of the participants. Of the 269 participants, 112 were men (63% controls and 37% with type 2 diabetes), and 157 were women (65% controls and 35% with type 2 diabetes). The mean age was significantly higher in participants with type 2 diabetes (55 ± 9 vs. control 39 ± 13 years old). Patients with type 2 diabetes were obese (BMI 37.5 ± 7.4 kg/m^2^), whereas control participants were overweight (28 ± 7.9 kg/m^2^). Patients with type 2 diabetes exhibited higher glucose levels, HbA1c (7.6 ± 2% for subjects with diabetes), and ACR (76 ± 79 mg/g). The average eGFR was significantly lower (83 ± 26 vs. control 95 ± 21 mL/min/1.73m^2^; *P* = 0.01) and creatinine significantly higher (0.5 ± 0.2 vs. control 0.7 ± 0.4 mg/L; *P* = 0.001), in participants with type 2 diabetes. Anti-hypertensive medications among of participants with type 2 diabetes include angiotensin receptors blockers (ARBs), angiotensin-converting enzyme inhibitors (ACEi), β-adrenergic blockers, calcium channel blockers, or diuretics. Patients with type 2 diabetes were on treatment with diabetic medications consisting of (combination of) insulin, metformin, dipeptidyl peptidase-4 inhibitors, glucagon-like peptide 1 receptor agonist, sulfonylureas, and thiazolidinediones.

**Table 1:** Demographic, clinical characteristics, demographic, and medications

Variables	Total participants		Men		Women	
	Control	Type 2 diabetes	Control	Type 2 diabetes	Control	Type 2 diabetes
Participants, *n* (%)	173 (64)	96 (36)	71 (63)	41 (37)	102 (65)	55 (35)
Age	39 (13)	55 (9)	40 (13)	56 (8)	39 (13)	55 (10)
Caucasians, *n* (%)	114 (42)	44 (16)	44 (16)	19 (7)	70 (26)	25 (9)
African American, *n* (%)	59 (22)	52 (20)	27 (55)	22 (45)	32 (51)	30 (49)
**Clinical analysis**						
BMI (kg/m^2^)	28 (7.9)	37.5 (7.4)	28 (6.9)	38 (7)	28 (8.6)	37 (7.8)
WHR	0.8 (0.1)	0.9 (0.1)	0.91 (0.08)	1.0 (0.05)	0.81 (0.07)	0.89 (0.1)
SBP (mm Hg)	118 (15)	132 (22)	121(14)	133 (25)	116 (15)	131 (20)
DBP (mm Hg)	74 (11)	80 (12)	74 (12)	80 (14)	73 (12)	80 (12)
Fasting glucose (mg/dL)	77 (19)	134 (54)	82 (18)	150 (54)	75 (19)	122 (51)
HbA1C (%)	–	7.6 (1.7)	–	8.3 (2)	–	7.2 (1)
Serum creatinine (mg/dL)	0.85 (0.1)	1.2 (1.9)	0.98 (0.15)	1.67 (2.9)	0.75 (0.1)	0.83 (0.2)
ACR (mg albumin/gCr)	28 (26)	76 (79)	27 (29)	78 (77)	29 (23)	74 (82)
uCreatinine (mg/L)	0.7 (0.4)	0.5 (0.2)	0.7 (0.4)	0.6 (0.2)	0.7 (0.4)	0.5 (0.2)
eGFR (mL/min per 1.73 m^2^)	95 (21)	83 (26)	94 (17)	77 (28)	95 (23)	87 (24)
**Medications**						
No meds, *n* (%)	169 (63)	25 (9)	69 (62)	10 (9)	99 (63)	15 (9)
ARB, *n* (%)	–	13 (5)	–	5 (5)	–	8 (5)
ACEi, *n* (%)	2 (1)	30 (11)	2 (2)	12 (10)	3 (2)	18 (11)
ARB/ACEi, *n* (%)	–	4 (1)	–	3 (3)	–	1 (1)
β-adrenergic blockers, *n* (%)	2 (1)	19 (7)	–	11 (10)	–	8 (5)
Calcium channel blockers, *n* (%)	–	1 (1)	–	–	–	1 (1)
Diuretics, *n* (%)	–	4 (2)	–	–	–	4 (3)
Diabetes medication, *n* (%)	1 (0.4)	80 (30)	1 (1)	35 (32)	–	46 (29)

Data are expressed as mean (SD). *ARB* angiotensin-II type 1 receptor blocker, *ACEi* angiotensin-converting I enzyme inhibitors. Anti-diabetic medications include insulin, metformin, dipeptidyl peptidase-4 inhibitors, glucagon-like peptide 1 receptor agonist, sulfonylureas, and thiazolidinediones

### Soluble PRR levels, renin activity, and uAGT levels and excretion display sex differences in patients with type 2 diabetes

Table 2 summarizes the levels of plasma and urine concentrations of sPRR, renin activity, and urine prorenin amount and urinary excretion of AGT and TGF-β1 in men and women from both groups.

**Table 2:** Soluble PRR, renin, prorenin, AGT, and TGF-β1 in plasma and urine

Variables	Total participants			Men			Women		
	Control	Type 2 diabetes	***P*** value	Control	Type 2 diabetes	***P*** value	Control	Type 2 diabetes	***P*** value
***Plasma***									
sPRR (ng/mL)	16.5 (15.8–17)	19.2 (17–21)^*^	**0.001**	18 (16.5–19)	17 (16–20)	0.87	15 (14.8–16) †	20 (18–22)‡	^**†**^**0.05** ^**‡**^**0.001**
Renin activity (ng Ang-I /mL/h)	3.2 (2.7–3.8)	12 (10–14)^*^	**0.001**	3.5 (2.5–4.5)	9.8 (6.4–13.3)^† ‡ **^	^**†**^**0.001** ^**‡**^**0.001** ^**^0.05	3.0 (2.3–3.7)	13 (11–15.6)^† ‡^	^**†**^**0.001** ^**‡**^**0.001**
***Urine***									
sPRR/uCr (ng/mg)	1.6 (1.3–1.8)	0.8 (0.6–1)^*^	**0.001**	1.8 (1.4–2.4)	0.6 (0.4–0.8)^† ‡^	^**†**^**0.001** ^**‡**^**0.05**	1.3 (1–1.7)	1.0 (0.75–1.3) †	^**†**^**0.001**
Renin activity (ng Ang-I /mL/h)	27 (21–33)	99 (77–120)^*^	**0.001**	27 (16.5–37.4)	125 (82–168)^† ‡ **^	^**†**^**0.001** ^**‡**^**0.001** ^**^0.05	27 (20–34)	84 (60–108)^† ‡^	^**†**^**0.001** ^**‡**^**0.001**
Prorenin/uCr (ng/g)	141 (109–173)	302 (232–371)^*^	**0.01**	128 (91–165)	362 (215–508)^† ‡^	^**†**^**0.01** ^**‡**^**0.05**	153 (103–204)	273 (199–348)^†^	^**†**^**0.01**
AGT/uCr (μg/g)	16 (13–19)	25 (17–32)^*^	**0.02**	15 (11–20)	30 (17–42)^† ‡^	^**†**^**0.05** ^**‡**^**0.05**	16 (12–20)	21 (11–20)	0.52
TGF-β1/uCr (pg/mg)	18 (14–22)	31 (24–38)^*^	**0.001**	16 (11–21)	33 (20–45)^†^	^**†**^**0.05**	19 (13–25)	31 (23–39)^†^	^**†**^**0.05**

Data are shown as mean (95% confident intervals). ^*^Different from total participants’ control; ^†^Different from men control; ^‡^Different from women control. ^**^Different from women with type 2 diabetes

#### Plasma and urine sPRR

Plasma sPRR was significantly elevated in participants with type 2 diabetes and to a greater extent in men compared to women, albeit by a small, but statistically significant, amount. Notably, plasma sPRR did not differ between male controls and subjects with diabetes. However, plasma sPRR concentration was augmented in women with type 2 diabetes compared to controls. In contrast, urinary concentrations of sPRR were significantly lower in diabetics, in both men and women compared to controls (Table 2).

#### Renin activity in plasma and urine

Plasma renin activity (PRA) was significantly higher in participants with type 2 diabetes compared to controls. In men and women analyzed separately, PRA was significantly increased in subjects with diabetes compared to controls and was higher in men than in women. Similarly, the renin activity was increased in the urine of subjects with type 2 diabetes compared to controls. Both women and men showed increased urine renin activity; however, the increase was greater in men than in women with type 2 diabetes (Table 2).

#### Prorenin amount in urine

After ten-fold concentration, prorenin amount in urine was measured and detected in 63% of controls and 84% of patients with type 2 diabetes. Prorenin was augmented in the urine of subjects with type 2 diabetes compared to controls. Although the concentrations of prorenin did not display sex differences between controls from both sexes, in subjects with type 2 diabetes the urine prorenin concentration was greater in men than in women (Table 2).

#### Urinary AGT excretion (uAGT)

The positive correlation between increased intrarenal RAS activity and risk of CKD has been indicated by the increases in uAGT of human subjects [2, 31]. In this cohort, men with type 2 diabetes exhibited significantly higher uAGT than controls, whereas no significant differences were found between women with type 2 diabetes (Table 2).

#### Urinary TGF-β1 excretion

Previous studies have indicated that prorenin-dependent activation of PRR bound to cell membranes may contribute to the stimulation of downstream signals as TGF-β1 [14]. Therefore, we quantified the levels of TGF-β1 in the urine. TGF-β1 was increased in patients with type 2 diabetes compared to controls. There were no sex differences observed among groups (Table 2).

### Correlations between sPRR concentrations and markers of renal function

Age, BMI, and PRA were positively correlated with plasma sPRR in all participants. However, when analyzed by sex, this correlation remained significant only for women. In addition, eGFR displayed was negatively correlated with plasma sPRR in women, but not in men (Table 3).

**Table 3:** Correlations between plasma and urine sPRR and risk factors for type 2 diabetes in participants

Variables	Total participants			Men			Women		
	***r***	***P value***	***n***	***r***	***P value***	***n***	***r***	***P value***	***n***
***Plasma***									
Age	**0.181**	**0.001**	244	0.102	0.311	101	**0.224**	**0.007**	143
BMI	**0.150**	**0.020**	244	0.097	0.334	101	**0.179**	**0.032**	143
DBP	0.067	0.3003	240	0.121	0.231	99	0.031	0.723	141
SBP	0.008	0.0866	240	0.081	0.424	99	0.080	0.343	141
Glucose	0.084	0.1997	233	0.002	0.983	97	0.132	0.124	136
HbA1c	− 0.087	0.464	80	− 0.204	0.278	37	0.032	0.838	42
Plasma renin activity	**0.273**	**< 0.001**	187	0.145	0.222	72	**0.499**	**< 0.001**	115
Urine renin activity	0.114	0.1104	196	0.171	0.135	78	0.071	0.442	118
ACR	0.061	0.3543	232	0.084	0.418	95	− 0.068	0.443	129
eGFR	− **0.147**	**0.024**	233	0.033	0.745	97	− **0.235**	**0.005**	137
***Urine***									
Age	− **0.240**	**< 0.001**	233	− **0.433**	**< 0.001**	101	− 0.094	0.282	132
BMI	− 0.109	0.0969	233	− **0.259**	**0.001**	101	0.014	0.873	132
DBP	− 0.124	0.0607	230	− 0.149	0.140	99	− 0.109	0.215	131
SBP	− 0.052	0.4251	230	− 0.033	0.742	99	− 0.081	0.354	131
Glucose	− 0.266	< 0.001	220	− **0.404**	**< 0.001**	96	− 0.137	0.127	124
HbA1c	− 0.011	0.924	78	− 0.053	0.773	32	0.119	0.429	46
Plasma renin activity	− 0.0853	0.2660	173	− 0.007	0.951	71	− 0.124	0.213	102
Urine renin activity	− **0.310**	**< 0.001**	185	− **0.330**	**< 0.001**	78	− **0.292**	**0.002**	108
ACR	− **0.140**	**0.036**	222	− 0.157	0.130	94	0.085	0.357	120
eGFR	**0.183**	**0.006**	221	**0.276**	**0.006**	96	0.113	0.208	125
TFG-β	0.081	0.371	123	− 0.193	0.188	48	**0.310**	**0.007**	75

In the entire cohort, urine sPRR was negatively correlated with age and ACR, but when this correlation was analyzed by sex, it remained significant only in men. Likewise, urine renin activity was negatively correlated with sPRR in urine of men and women from the control group. Additionally, urine sPRR was negatively correlated with age, fasting glucose, and eGFR in men. However, in women, a positive correlation was only observed between sPRR levels and TGF-β1 in urine. HbA1c did not correlate with sPRR concentrations in plasma and urine.

### eGFR rate is associated with sPRR levels and renin activity in plasma and urine

Multiple linear regression analyses in all the participants were performed to determine if the relationship between plasma and urine sPRR levels, renin activity, and eGFR (Table 4). Model 1 represents the simple regression analysis between eGFR, urine renin activity, and sPRR in plasma and urine. Model 2 represents the relationships between eGFR and levels of sPRR in plasma and urine after adjusting for diabetes, age, sex, race, body weight, BMI, DBP, SBP, anti-hypertensives, and diabetic medication. Using these two models, we found a significant association between eGFR, plasma and urine sPRR, urine renin activity, and age, race, and anti-hypertensive. Also, body weight was significantly associated in urine renin activity and urine sPRR.

**Table 4:** Multiple regression analysis with eGFR

Predictors	Urine Renin Activity			Plasma sPRR			Urine sPRR		
	***β***	***t***	***P*** value	***β***	***t***	***P*** value	***β***	***t***	***P*** value
Model 1—constant	94.47	49.29	< 0.001	100.52	20.55	< 0.001	86.11	41.58	< 0.001
Model 2—constant	114.89	9.61	< 0.001	117.29	10.18	< 0.001	112.37	9.88	< 0.001
Type 2 diabetes	− 3.79	− 0.89	0.377	− 5.19	− 1.39	0.1673	− 2.76	− 0.74	0.462
Age	− 0.70	− 5.75	**< 0.001**	− 0.72	− 6.25	**< 0.001**	− 0.688	− 5.51	**< 0.001**
Sex	3.09	1.02	0.307	3.81	1.40	0.1640	3.73	1.31	0.190
Race	− 9.66	− 3.05	**0.0026**	− 11.67	− 4.01	**< 0.001**	− 11.89	− 4.01	**< 0.001**
Body weight (kg)	− 0.06	− 0.18	**0.010**	− 0.486	− 1.83	0.073	2.69	2.52	**0.012**
BMI	0.33	1.68	0.093	0.197	1.08	0.2829	0.17	0.92	0.359
DBP	0.137	0.90	0.371	0.154	1.06	0.2887	0.22	1.42	0.158
SBP	− 0.05	− 0.53	0.594	− 0.04	− 0.40	0.6877	− 0.09	− 0.92	0.359
Anti-hypertensives	− 1.18	− 1.96	0.052	− 1.751	− 3.254	**0.001**	− 2.10	− 3.94	**< 0.001**
Anti-diabetic medications	1.84	0.29	0.773	3.762	0.639	0.524	0.85	6.11	0.900

Urine Renin Activity: Model 1: *P =* 0.0038, *R*^2^ = 3.9%; Model 2: *P* < 0.001, *R*^2^ = 24.32%

Plasma sPRR: Model 1: *P* = 0.0058, *R*^2^ = 3.4%; Model 2: *P* < 0.001, *R*^2^ = 25.67%

Urine sPRR Model 1: *P* = 0.0357, *R*^2^ = 3.4%; Model 2: *P =* 0.001, *R*^2^ = 26.61%

### Sex effect on renin activity, sPRR, and prorenin in patient with type 2 diabetes by anti-hypertensive treatment

The effects of anti-hypertensive treatments on sPRR, renin activity, and prorenin varied in the patients of this study. In patients with type 2 diabetes, treatment with ARBs only increased plasma sPRR in women, whereas ACEi increased PRA primarily in men. In contrast, regardless that all patients with type 2 diabetes treated with β-blockers displayed increased PRA, plasma sPRR was augmented only in women, but not in men.

In addition, despite anti-hypertensive treatment, no changes were found in urine sPRR in patients with type 2 diabetes. Urine renin activity was increased among men and women with type 2 diabetes treated with blood pressure medications. Interestingly, beta-blockers increased urine prorenin in diabetic men, but not in women counterpart. Furthermore, in these patients, the combined treatment with ACEi and ARBs was accompanied by increased urine prorenin only in men, whereas ACEi increased urine prorenin in women (Table 5).

**Table 5:** Soluble PRR, renin, and prorenin in plasma and urine distributed by type of anti-hypertensive treatment

	Control	Type 2 diabetes						
		No meds	ARBs	ACEi	ARB/ACEi	β-blockers	Calcium blockers	Diuretics
Total participants***, n***	167	25	13	30	4	19	1	4
***Plasma***								
sPRR (ng/mL)	16.6 ± 4.1	17.3 ± 6.4	21.8 ± 9.8*	18.1 ± 4.3	23.3 ± 7.0	20.0 ± 8.6	11.9	22.7 ± 4.1
Renin activity (ng Ang-I/mL/h)	3.3 ± 0.3	13.2 ± 2.0*	17.1 ± 1.7*	11.8 ± 1.7*	5.4 ± 1.2	10.0 ± 1.7*	21.12	7.5 ± 5.5
***Urine***								
sPRR/uCr (ng/mg)	16 ± 1.6	0.95 ± 0.8	0.90 ± 0.8	0.81 ± 0.9	0.40 ± 0.2	1.0 ± 1.4	115	0.91 ± 0.5
Renin activity (ng Ang-I/mL/h)	26.9 ± 36	102.1 ± 102*	51.46 ± 44*	96.89 ± 105*	221.1 ± 182*	102.2 ± 73*	273.8	50.12 ± 23*
Prorenin/uCr (ng/g)	142.1 ± 159	270.7 ± 326	346.5 ± 281.8	275.5 ± 236.1	113.0 ± 12.87	357 ± 336*	1075	202 ± 148
**Men,** ***n***	**69**	**10**	**5**	**12**	**3**	**11**	**0**	**0**
***Plasma***								
sPRR (ng/mL)	18 ± 4.7	16.3 ± 5.0	17.4 ± 4.8	18.2 ± 4.7	25.4 ± 7.0	17.0 ± 6.2	–	–
Renin activity (ng Ang-I/mL/h)	3.2 ± 3.6	13.2 ± 12*	4.2 (n = 1)	9.3 ± 7.1^†^	4.9 ± 2.8	6.5 ± 3.3	–	–
***Urine***								
sPRR/uCr (ng/mg)	1.4 ± 1.2	0.78 ± 0.8	0.4 ± 0.5	0.65 ± 0.5	0.36 ± 0.25	0.65 ± 0.5	–	–
Renin activity (ng Ang-I/mL/h)	15 ± 17.6	131.5 ± 1.4*	28.41 (n = 1)	108 ± 107*	308 ± 142*^†^	105.8 ± 52*	–	–
Prorenin/uCr (ng/g)	120 ± 97.2	380 ± 0.4	953.5 (n = 1)	215 ± 156	113 ± 12.9^‡^	520.2 ± 388*	–	–
**Women,** ***n***	**99**	**15**	**8**	**18**	**1**	**8**	**1**	**4**
***Plasma***								
sPRR (ng/mL)	15.5 ± 3.3	17.8 ± 7.3	24.5 ± 11.3*^†^	18.1 ± 4.2	17.2	23.8 ± 10*^†^	12	22.7 ± 4.2
Renin activity (ng Ang-I/mL/h)	2.8 ± 2.4	13.2 ± 8.0*	18.8 ± 1.9*	13.3 ± 8.4*	6.4	11.1 ± 6.4*	21.12	7.5 ± 9.7^‡^
***Urine***								
sPRR/uCr (ng/mg)	0.95 ± 0.08	0.86 ± 0.1	1.2 ± 0.1	0.49 ± 0.1	0.53	1.5 ± 0.2	0.11	0.780 ± 0.61
Renin activity (ng Ang-I/mL/h)	19.5 ± 22	84 ± 68*	54 ± 45	67.6 ± 50*	46	97.6 ± 98*	273	50.1 ± 23
Prorenin/uCr (ng/g)	99.4 ± 84.7	207 ± 198	260 ± 150	312.3 ± 271*	–	193.4 ± 180	1075	202 ± 147

Data are expressed as mean ± SD. *sPRR* soluble prorenin receptor, *uCr* urine creatinine. *Different from controls; Different from patient with type 2 diabetes: ^†^no medication, ^‡^ACEi, ^‡^ABRs

### Urine renin activity is an indicator of renal dysfunction in patients with type 2 diabetes

Urinary excretion of AGT has been proposed as a biomarker of intrarenal RAS activation in patients with type 2 diabetes [2]. We further examined whether renin activity in the urine could be also considered a potential indicator of intrarenal RAS stimulation. Our results indicated that in patients with type 2 diabetes, urine renin activity increased as the concurrence of diabetes status, hypertension, eGFR < 60 mL/min per 1.73 m^2^, and/or ACR also increased (Supplemental*,* Figure S1).

### Augmentation of uAGT is associated with high levels of sPRR in men but not women

We further investigated the relationship between plasma sPRR and uAGT. Plasma sPRR did not correlate with uAGT excretion in all participants (Supplemental Figure S2A). However, when patients were divided into low plasma sPRR (≤ 16 ng/mL) and high plasma sPRR (≥ 19 ng/mL) groups, uAGT was significantly higher in those patients with greater levels of plasma sPRR than in patients with low levels (Supplemental Figure S2B). This difference was significant only in men (*P* < 0.02), but not in women (*P* = 0.73; Supplemental*,* Figure S2C).

## Discussion

The present study reveals important new findings regarding sex differences in the sPRR in patients with type 2 diabetes. (1) Patients with type 2 diabetes exhibit sex differences in sPRR concentrations in plasma and urine: (*i*) Plasma sPRR concentration is higher in women compared to men; (*ii*) Plasma sPRR correlates with age, BMI, eGFR, and PRA in women, but not in men; (*iii*) In contrast to plasma, urine sPRR concentrations are markedly decreased in both men and women. (2) Urine sPRR correlates with renin activity in both men and women; but it only correlates with age, BMI, glycemia, and eGFR in men, not in women. Our results also indicate that eGFR is associated with sPRR in plasma and urine, as well as with renin activity in urine after adjusting by age and race. (3) Renin activity in urine increases with the co-existence of increased ACR, hypertension, and reduced eGFR. (4) The effects of anti-hypertensive treatment on sPRR differ by sex: ARBs and β-blockers increase plasma sPRR in women but not in men, whereas ACEis increase PRA but not sPRR. In contrast, increase plasma sPRR was augmented only in women, but not in men. These findings have important clinical implications due to the potential role of sPRR as indicator of intrarenal RAS activation and renal dysfunction in men and women with type 2 diabetes.

Sex is a relevant biological variable in the prevalence and pathophysiology of CV diseases [28, 32]. Women with type 2 diabetes experience a disproportionately greater CV burden than men [4]. Interestingly, estrogens are beneficial to glucose homeostasis, and clinical trials also suggest that early menopause is associated with a higher risk of type 2 diabetes development compared to the average menopausal age [27]. Our results unveil the existence of a sexual dimorphism in the plasma concentrations of sPRR in healthy subjects and patients with type 2 diabetes. In this study, plasma sPRR and PRA were both significantly higher in women with type 2 diabetes as compared with their male counterparts. The mechanism by which estrogens increase plasma sPRR in postmenopausal women is not clear. It is possible that an interaction with the G protein-coupled estrogen receptor (GPER) plays a significant role. Resveratrol, which can activate GPER [33], decreases PRR expression, profibrotic and pro-inflammatory proteins, and vascular remodeling in aging mice [34]. However, it has not been established whether GPER is protective in women with type 2 diabetes by decreasing circulating sPRR and PRR-mediated CV alterations. Current studies are undergoing to clarify these issues. In different cohorts, plasma sPRR levels do not vary by sex, age, circadian rhythms, hormones, or diabetes status [22, 26]. However, in our study, plasma sPRR concentrations differed between men and women, particularly among subjects with diabetes. Most of our patients with type 2 diabetes were hypertensive, obese, or middle-aged. In women with essential hypertension, plasma sPRR correlates with age [22]. Full-length PRR is physically associated with the vacuolar H^+^-ATPase (V-ATPase) and traffics to the cell surface, whereas the sPRR is secreted to the extracellular compartment [16–18]. It is possible that plasma sPRR is increased in patients at middle age as a mechanism of compensation of PRR due to V-ATPase dysfunction and impaired autophagy during aging [22, 35]. Although in our study we did not measure plasma estrogen levels neither collect information on different phases of the menstrual cycle, it is possible that sex hormones may regulate sPRR in plasma and CV complications in subjects with type 2 diabetes [22, 26, 36]. The fact that women with type 2 diabetes exhibit decreased protection from estrogens is due, in part, to maladaptive RAS activation [4, 28], suggest that careful considerations should be given to increased levels of sPRR in the plasma, particularly in postmenopausal women because they are at higher risk of CV complications and development of diabetic kidney disease and end-stage kidney disease [28, 37]. Similarly, prorenin is increased in the plasma of patients with type 2 diabetes, which is associated with the presence of microalbuminuria [29] and the onset of CV complications [38]. Previous studies have shown that plasma concentrations of prorenin and renin are elevated in obese women not receiving estrogen therapy when compared with those of postmenopausal women treated with hormone therapy [39]. However, whether sPRR contributes to the activation of prorenin in vivo has remained controversial [11, 40]. More studies including subjects with CV and kidney diseases are needed to better define the clinical impact of plasma sPRR in CV complications, particularly in women with type 2 diabetes.

The concentrations of sPRR in the urine of patients with type 2 diabetes differ between men and women and may reflect local changes of intrarenal RAS activation. Elevated plasma sPRR levels may reflect decreased clearance of sPRR from the kidney [22]; since sPRR is a small molecule (28 kDa) compared with albumin, it is easily filtered in the glomerulus, and plasma and urine concentrations should be in equilibrium. However, whether urinary sPRR is excreted without being absorbed from the proximal tubules is unclear. In the present study, we attempted to analyze the relationship between sPRR in urine and eGFR after correcting by risk factors of type 2 diabetes (Table 3). The demonstration that sPRR in urine-positive correlates with eGFR in men but not in women suggest that urine sPRR in men with type 2 diabetes might be associated with the stimulation of urine renin activity and eGFR decline over time. Although patients with essential hypertension seem to not display sexual dimorphisms in this regard, Ichihara and associates reported that plasma sPRR correlates positively with renal function independent of age, BP, and HbA1c [22]. These results support the fact that in patients with type 2 diabetes, the pathophysiology of renal injury is sexually dimorphic; because although women with type 2 diabetes are more susceptible to changes in eGFR and progression of end-stage kidney disease, the overall progression of the disease slows down compared to men [29]. The underlying mechanism of this relationship is not clear and requires further investigation. Multiple regression analysis revealed that there is a significant association between eGFR with urine renin activity, and plasma and urine sPRR levels. In addition, we found a significant association between age, race, and anti-hypertensives with eGFR, which support the concept that eGFR declines with age and that African American participants have on average a decreased eGFR compared to Caucasians after adjusting for urine sPRR, diabetes mellitus status, age, sex, BMI, DBP, SBP, and medications. Interestingly, only men with type 2 diabetes displayed differences in renal dysfunction parameters which were correlated with urine sPRR. Urinary excretion of sPRR was negatively associated with age, BMI, and blood glucose in men, but not in women. However, HbA1c did not correlate with plasma and urine sPRR levels in patients with type 2 diabetes. Sautin et al. demonstrated that glucose promotes V-ATPase trafficking to plasma membrane in the renal epithelial cells [41]. Thus, these results suggest that glucose can stimulate the trafficking PRR/V-ATPase to the plasma membrane.

The urinary TGF-β1 excretion is augmented in patients with type 2 diabetes and is associated with the progression of renal failure [42, 43]. These findings might be relevant to our study. It has been reported that the activation of PRR membrane-bound isoform by renin and prorenin stimulates TGF-β1 [14]. In diabetes, the collecting duct is the main supplier of prorenin [44]. Here, we showed that in patients with type 2 diabetes, prorenin levels were increased in urine samples concentrated ten-fold before assaying. Given that prorenin is produced in large amounts by the collecting duct in diabetes [44], it is likely that PRR bound to cell plasma membranes is activated by locally produced prorenin, which could explain the increases in TGF-β1 in urine during the progression of renal disease in the diabetic kidney. Future studies are warranted to examine the underlying mechanisms explaining the dissociation between plasma and urine sPRR concentrations in men and women with type 2 diabetes, and the impact on the stimulation of pro-inflammatory factors in kidney disease.

The activation of PRR in renal tubules contributes to the development of kidney injury in two different ways: directly via the stimulation of intracellular signals linked to fibrosis and inflammation [5, 8, 9, 14, 15], and indirectly by leading to further formation of intrarenal/intratubular angiotensin-II (Ang-II) [14, 45, 46]. In mice with specific PRR deficiency in the collecting duct (CD), PRR for activation of locally generated prorenin, increases in renin activity, and contributes to the formation of *de novo* Ang-II in the lumen of the CD [46]. In the present study, patients with type 2 diabetes had greater increases in renin activity in the urine. Urine renin activity may reflect the severity of kidney dysfunction, which emphasizes its importance as a potential marker of maladaptive intrarenal/intratubular RAS activation in patients with type 2 diabetes. The fact that prorenin has been reported as undetectable in the urine [47, 48] is consistent with reabsorption of renin by the proximal tubules [49, 50] and that renin in urine does not reflect prorenin conversion to renin in the intratubular compartment [50, 51]. Unfortunately, most of the studies reporting renin levels in urine utilize commercially available ELISA kits, which rely on prorenin as the standard provided. In these studies, renin levels in urine are reported at least ten-fold higher than those measured and referenced by the International Reference Preparation of human renin [52]. In the present study, the fact that renin activity measured by radioimmunoassay was increased in urine four- and three-fold in men and women with type 2 diabetes, respectively, suggests that active renin was probably generated further down in the distal nephron segments. Recent studies reported that megalin reabsorbs renin in the proximal tubules since megalin knockout mice exhibited excessive urinary excretion of renin [49, 50]. Megalin is primarily expressed in the apical brush border of the proximal tubules and contributes to reabsorption of filtered molecules [53]. Impairment of this process is the hallmark of the many forms of renal disorders, including DN [53]. Therefore, we cannot rule out that the augmentation of renin activity and prorenin in the urine of patients with type 2 diabetes is due to altered glomerular filtration and/or impaired proximal tubular reabsorption.

Anti-hypertensive treatments on sPRR, renin activity, and urine prorenin differ between men and women with type 2 diabetes. Renin activity in plasma increased by anti-hypertensive medications in subjects with diabetes [47]. In the present study, the fact that ARBs increase in plasma sPRR parallel augmentation of PRA support further support the concept that plasma sPRR is an indicator of systemic RAS activation in women with type 2 diabetes. In contrast, although diuretics increased renin activity in plasma and urine of patients with type 2 diabetes, primarily in women; these findings were not associated with changes in sPRR. Systemic and intrarenal RAS activation in patients with type 2 diabetes and hypertension may differ between men and women [54]. However, most studies focused on CV and renal responses to RAS blockade examine primarily male subjects, and studies including humans or animal of both sexes are scarce [55]. However, a few studies report reduced effectiveness of ARBs and ACEi in females. Modeling and analyses from male and female normotensive and hypertensive rats shows that ACEi and ARBs induce similar decreases in AT1R-bound Ang-II when expressed as percent change instead of absolute values [56]. Likewise, mathematical models predict preferential beneficial vasodilatory effects in women after treatment with ARBs as compared to ACEi, likely mediated via AT2R [57]. Further studies are required to elucidate the mechanisms underlying on the sex differences in the efficacy of anti-hypertensives.

We did not quantify the levels of Ang-II in urine, but the concomitant augmentation of renin activity and uAGT, particularly from men with type 2 diabetes, emphasizes the importance of intrarenal and intratubular RAS activation in the progression of DN and CKD [31]. Indeed, the inhibition of sodium-glucose cotransporter prevents the augmentation of intrarenal AGT and uAGT by mitigating renal tubular fibrosis and inflammation in mice [58] and humans [59] with type 2 diabetes. Although in the present study uAGT did not correlate with plasma sPRR in total participants, it did in men with type 2 diabetes. Furthermore, the fact that PRR is involved in the progression of mesangial fibrosis in patients with diabetic kidney disease [60] highlights the relevance of plasma sPRR as a potential biomarker of CKD. Clinical trials documenting that ACEi and ARBs treatments only slow down, but do not halt, the progression of CKD among patients with diabetic or non-DN [61] demonstrates the critical need for novel therapeutic targets in renal disease. Future studies are warranted to define the relevance of sPRR in the pathogenesis of CKD and its impact as a clinical biomarker for the identification of patients at a high risk for CKD.

### Limitations

Our study has some limitations. Patients with type 2 diabetes were older and had higher BMI than control participants. Both age and obesity might influence the concentrations of sPRR in the plasma. Unfortunately, data on duration of hypertension and diabetes, collected information on different phases of menstrual cycle, menopause status, estrogen replacement therapy, and HbA1c levels, which were evaluated only in participants with the diagnosis of diabetes not in controls subjects, are not available. Further studies are warranted to measure sPRR at different time-points in pre- and peri-menopause women and younger men in order to elucidate if plasma and urine sPRR concentrations could be used as predictors for the systemic and intrarenal RAS status, type 2 diabetes, and CKD onset and progression in men and women later in life. These studies will help to define the effects of sex hormones on plasma and urine sPRR concentrations and their impact on clinical outcomes in CVD.

## Conclusions

Patients with type 2 diabetes exhibit sex differences in sPRR concentrations in plasma and urine. In diabetic women, increased plasma sPRR parallels the activation of systemic RAS; while in diabetic men, augmentation of sPRR in urine matches with intrarenal RAS stimulation. In patients with CV disease, sPRR might be a potential indicator of intrarenal RAS activation and renal dysfunction in men and women with type 2 diabetes.

### Perspectives and significance

In patients with type 2 diabetes, high circulating levels of prorenin are associated with the onset of microalbuminuria and the occurrence of microvascular complications. The ability of sPRR to activate prorenin raises the possibility that the interactions between sPRR and prorenin in the systemic circulation, or within the kidney intratubular compartments, may contribute to either systemic or intrarenal RAS activation and progression of CKD in patients with type 2 diabetes. Data from the present study indicate that sPRR exhibit sexual dimorphisms in patients with type 2 diabetes. In these patients, increases in plasma sPRR parallel urine albumin/creatinine ratio, hypertension, and decline in renal function. Specifically, plasma sPRR concentration is higher in women as compared to men. In contrast to plasma, urine sPRR concentrations are markedly decreased in both men and women. Recently, it has been suggested that sPRR may exert beneficial effects on a rodent model of type 2 diabetes [62]. As stated by Yang and associates, the chronic infusion of recombinant sPRR-His in high-fat diet-induced type 2 diabetic mice decreased obesity, hyperglycemia, insulin resistance, and albuminuria. In this regard, since type 2 diabetes is a complex metabolic disease, it is possible that compensatory mechanisms counter sex and hormone effects by the increased sPRR levels. Furthermore, aging must be an additional factor that might contribute to metabolic status in response to changes in plasma sPRR [22]. Identifying the underlying mechanisms of sex disparities of sPRR and clinical outcomes would yield unique insights on the impact of sPRR on CV risk and kidney dysfunction in patients with type 2 diabetes.

## Supplementary Information

**Additional file 1: Supplementary Figure S1.** Renin activity in urine by moderate loss of kidney function, hypertension (HTN) and albumin/creatinine (ACR: urine albumin to creatinine ratio). Data are expressed as mean ± Standard error of the mean (SEM). *P = 0.001 different from control group, † *P*<0.001 different from group 2 (type 2 diabetes and HTN), ‡ different from group 3 (type 2 diabetes, HTN and ACR). **Supplementary Figure S2.** (A) Correlation between soluble prorenin receptor (sPRR) levels and urinary angiotensinogen (uAGT) excretion (r = 0.05; *P* = 0.42). Data are expressed as mean ± SEM. Comparisons of uAGT between low (≤16 ng/mL) and high (≥19 ng/mL) levels of sPRR in patients with diabetes (B) and by gender (C). (C) *P < 0.02. (D) Men *P* = 0.03 and Women *P* = 0.73.

## Data Availability

The data and analyses of this study are available from the corresponding author on reasonable request.
