# Supplementary material for: Sex differences in soluble prorenin receptor in patients with type 2 diabetes
Source: Biol Sex Differ. 2021 May 1;12:33. doi: 10.1186/s13293-021-00374-3 (PMC8088668; doi:10.1186/s13293-021-00374-3)
Supplement: Supplementary file 1 — Additional file 1: Supplementary Figure S1. Renin activity in urine by moderate loss of kidney function, hypertension (HTN) and albumin/creatinine (ACR: urine albumin to creatinine ratio). Data are expressed as mean ± Standard error of the mean (SEM). *P = 0.001 different from control group, † P<0.001 different from group 2 (type 2 diabetes and HTN), ‡ different from group 3 (type 2 diabetes, HTN and ACR). Supplementary Figure S2. (A) Correlation between soluble prorenin receptor (sPRR) levels and urinary angiotensinogen (uAGT) excretion (r = 0.05; P = 0.42). Data are expressed as mean ± SEM. Comparisons of uAGT between low (≤16 ng/mL) and high (≥19 ng/mL) levels of sPRR in patients with diabetes (B) and by gender (C). (C) *P < 0.02. (D) Men P = 0.03 and Women P = 0.73. [file 13293_2021_374_MOESM1_ESM.docx]

**Supplemental Material**

**Sex Differences in Soluble Prorenin Receptor in Patients with Type 2 Diabetes**

***Bruna Visniauskas^1^; *Danielle Y. Arita^1^,** Carla B. Rosales^1^; Mohammed A. Feroz^1^; Christina Luffman^1^; Michael J. Accavitti^1^; Gabrielle Dawkins^1^; Jennifer Hong^1^; Andrew C. Curnow^1^; Tina K. Thethi^2,3^; John J. Lefante^4^; Edgar A. Jaimes^5^; Franck Mauvais-Jarvis^2,6,7^; Vivian A. Fonseca^2,6^; Minolfa C. Prieto^1,8^

**(*)** These authors contributed equally to this work as co-first authors

^1^Department of Physiology, Tulane University School of Medicine, New Orleans, LA, USA.

^2^Department of Medicine, Endocrinology Division, Tulane University School of Medicine, New Orleans, LA, USA.

^3^AdventHealth, Translational Research Institute, Orlando, FL, USA

^4^Department of Biostatistics and Data Science, School of Public Health and Tropical Medicine, New Orleans, LA, USA.

^5^Renal Service, Memorial Sloan Kettering Cancer Center, New York, NY, USA.

^6^Southeast Louisiana Veterans Healthcare System New Orleans, LA, USA.

^7^Tulane Center of Excellence in Sex-Based Biology & Medicine, New Orleans, LA, USA.

^8^Tulane Hypertension and Renal Center of Excellence, Tulane University School of Medicine New Orleans, LA, USA.

***** These authors contributed equally to this work as co-first authors

**Supplementary Figure S1.** Renin activity in urine by moderate loss of kidney function, hypertension (HTN) and albumin/creatinine (ACR: urine albumin to creatinine ratio). Data are expressed as mean ± Standard error of the mean (SEM). **P* = 0.001 different from control group, † *P*<0.001 different from group 2 (type 2 diabetes and HTN), ‡ different from group 3 (type 2 diabetes, HTN and ACR).

**
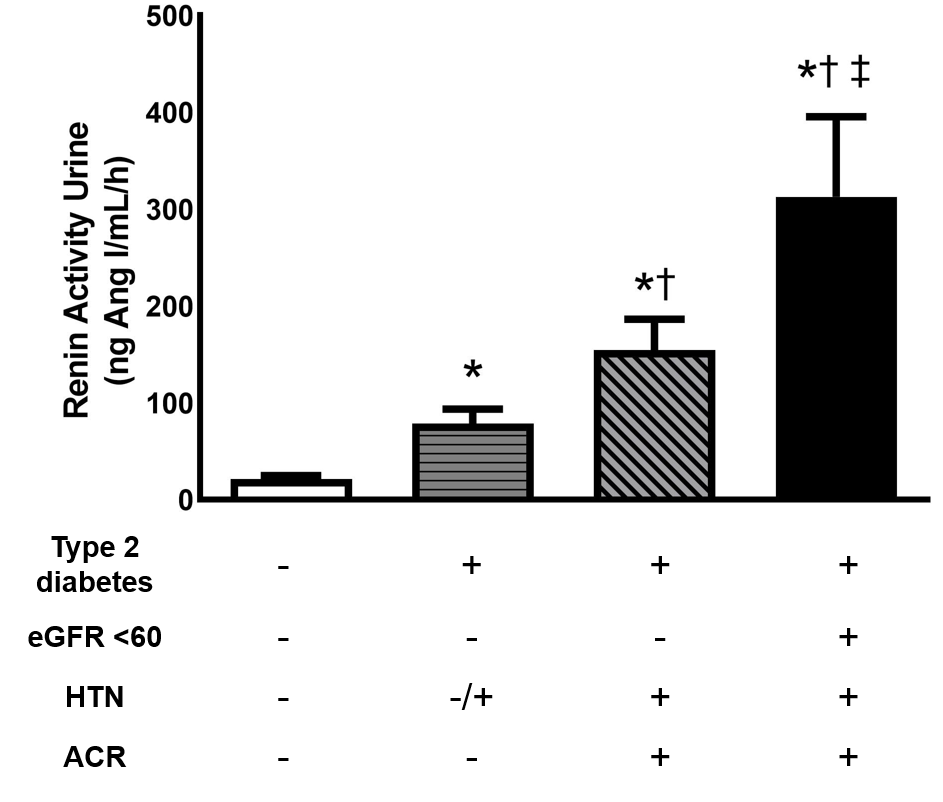
**

**
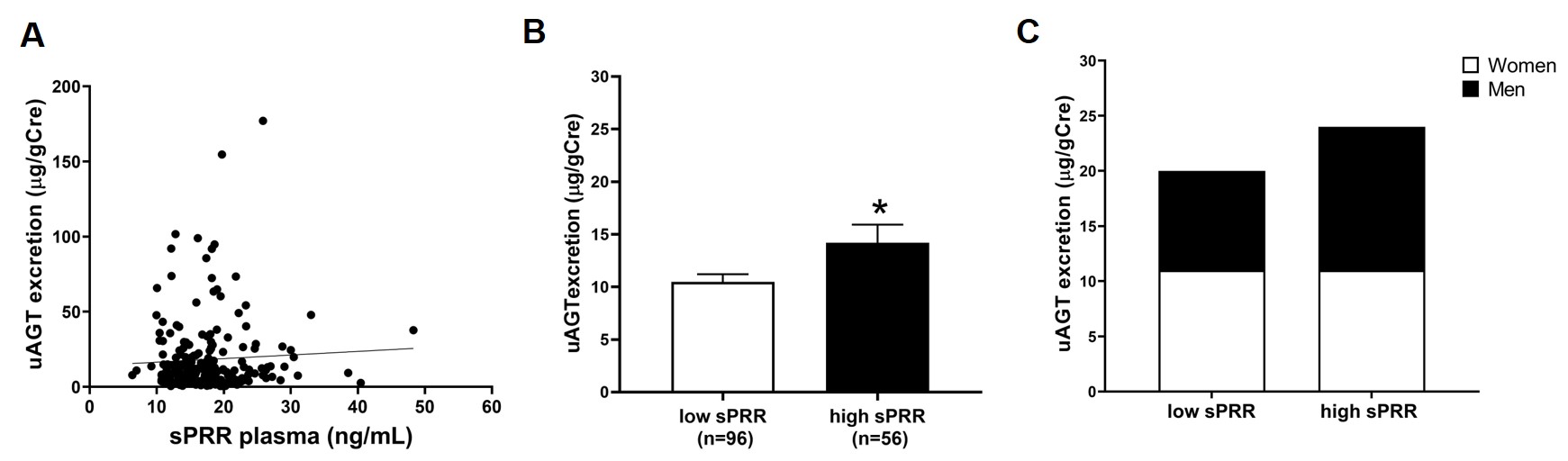
Supplementary Figure S2.** (A) Correlation between soluble prorenin receptor (sPRR) levels and urinary angiotensinogen (uAGT) excretion (r = 0.05; *P* = 0.42). Data are expressed as mean ± SEM. Comparisons of uAGT between low (≤16 ng/mL) and high (≥19 ng/mL) levels of sPRR in patients with diabetes (B) and by gender (C). (C) **P* < 0.02. (D) Men *P* = 0.03 and Women *P* = 0.73.
